# DO PHYSICAL DISABILITY, FOOD INSECURITY, AND NEIGHBORHOOD COHESION MATTER IN DEPRESSION?

**DOI:** 10.1093/geroni/igad104.3287

**Published:** 2023-12-21

**Authors:** Jihyeong Jeong, Paul Sacco

**Affiliations:** University of Maryland Baltimore, Baltimore, Maryland, United States; University of Maryland Baltimore, Baltimore, Maryland, United States

## Abstract

Although previous studies have explained the association between food insecurity and depression, there has been a dearth of studies that have examined this relationship with older adults with physical disabilities and explored other variables that could have potentially impacted this relationship. Given the social capital theory, recognizing community-level factors provides broader contexts about the physical and social environments of communities where older adults with physical disabilities live. Thus, this study aimed to explain how physical disability and food insecurity are related to depression and examine the buffering effect of neighborhood cohesion on the relationship between food insecurity and depression. We analyzed 5,668 older adults aged 60 years or over from the Health and Retirement Study 2020 Core Final Release. This study conducted logistic regressions to examine the buffering effects of neighborhood cohesion on the association between food insecurity and depression, including covariates. The results showed that physical disability and food insecurity were statistically significantly associated with depression. Interestingly, the hypothesis of the buffering effects of neighborhood cohesion was not supported. This result can be explained by characteristics of mental health that different factors beyond food insecurity and neighborhood cohesion may influence. In other words, neighborhood cohesion alone may not be able to explain its buffering effects on the relationship between food insecurity and depression. While the results of this study may seem contrary to social capital theory, future studies need to consider various moderating variables to understand how community and stressors such as food insecurity interact with mental health.

